# The relationship between digital health literacy and problematic mobile social media use among adolescents: the chain mediating role of physical activity and social–emotional competence

**DOI:** 10.3389/fpsyg.2026.1801252

**Published:** 2026-04-20

**Authors:** Chuyuan Zhao, Yingbo Zhu, Yu Zhang, Bihui Cui, Tianxing Liu, Xudong Shen, Shulin Yu

**Affiliations:** 1 School of Physical Education and Sport, Henan University, Kaifeng, China; 2 School of Physical Education and Sports Science, South China Normal University, Guangzhou, Guangdong, China; 3 Shanxi Gymnastics and Martial Arts Sports Center, Taiyuan, Shanxi, China; 4 Physical Education Department, Dalian Minzu University, Dalian, China; 5 Shenyang Sport University, Shenyang, China; 6 School of Athletic Training, Tianjin University of Sport, Tianjin, China

**Keywords:** adolescents, digital health literacy, physical activity, problematic mobile social media use, social–emotional competence

## Abstract

**Objective:**

With the rapid expansion of digital technologies and the global increase in social media use, this study aimed to investigate the relationship between digital health literacy (DHL) and problematic mobile social media use (PMSMU) among adolescents, as well as the underlying mechanisms.

**Methods:**

A total of 555 adolescents were surveyed using the eHealth Literacy Scale (eHEALS), the Problematic Mobile Social Media Usage Assessment Questionnaire, the Physical Activity Rating Scale (PARS-3), and the social–emotional competence scale.

**Results:**

DHL was significantly correlated with physical activity (PA) (*r* = 0.233, *p* < 0.001), social–emotional competence (SEC) (*r* = 0.133, *p* < 0.001), and PMSMU (*r* = −0.120, *p* < 0.001). Mediation analysis revealed three significant indirect pathways: PA mediated the relationship (21.43%), SEC mediated it (10.71%), and a sequential pathway through PA and SEC also emerged (5.36%).

**Conclusion:**

DHL not only directly predicts PMSMU but also indirectly predicts it through the independent and chain mediating effects of PA and SEC. Among these indirect pathways, PA emerged as the most influential mediator.

## 1 Introduction

With the widespread proliferation of smart devices, today’s adolescents have become typical “Digital Natives” (Prensky, 2001). This digital transformation is reflected globally, where social media use has continued to grow at a remarkable pace. According to *the Digital 2026 Global Overview Report*, there were 5.66 billion active social media user identities worldwide as of October 2025, representing 68.7% of the global population—an increase of 259 million users (4.8%) over the previous 12 months (World Health Organization, 2025b). This widespread use has heightened concerns about problematic social media use (PMSMU) and its potential impact on mental health, as highlighted by the World Health Organization (2025a). In China, a similar trend is evident. According to the country’s 5th National Survey on Internet Usage among Minors, as of 2023, the number of minor internet users exceeded 193 million, with an internet penetration rate of 97.2% (Organization, T.H, 2024). Notably, over 80% of adolescents access the internet weekly via mobile terminals such as smartphones and tablets (Organization, T.H, 2024). Against this backdrop, mobile social networks, characterized by their “always-on” connectivity, have profoundly reshaped adolescents’ modes of social interaction and emotional bonding (Prochnow et al., 2025). Thus, adolescent PMSMU has gradually evolved into a widely concerning public health issue (Fineberg et al., 2025). Distinct from traditional internet addiction, PMSMU emphasizes the characteristics of uncontrollable use within mobilized and contextualized scenarios, as well as the persistent impairment of individual social functioning (Billieux et al., 2015). Given that adolescence is a critical developmental period marked by social role reconstruction, intensified emotional fluctuations, and immature self-regulation capabilities, these physical and mental developmental characteristics render adolescents a highly susceptible population for PMSMU when facing highly immersive mobile social environments (Throuvala et al., 2019). Uses and Gratifications Theory (UGT) posits that individuals actively select and use media to satisfy specific psychological and social needs (Katz et al., 1973). From this perspective, adolescents’ mobile social media use can be understood as an active selection process aimed at fulfilling such needs (Heravi et al., 2018). Relevant data indicate that 58.1% of adolescents use mobile social networks to meet interpersonal interaction needs, 44.3% to alleviate loneliness, while 33.0 and 27.8% aim to achieve self-expression and obtain social recognition, respectively (Ciudad-Fernández et al., 2024). However, when emotional gratification in virtual scenarios gradually supplants real-world functioning, this need-oriented usage behavior may undergo a qualitative change, thereby evolving into uncontrollable PMSMU (Kardefelt-Winther, 2014). Existing evidence suggests that with the continuous increase in the time and intensity of mobile social media use among adolescents, it not only causes direct damage to physical health (e.g., sedentary behavior, sleep deprivation) but may also erode social–emotional functioning in real life, inducing a series of psychological and social adaptation problems (Alonzo et al., 2021; Valkenburg et al., 2022). Faced with this challenge, simple restrictive interventions often treat the symptoms rather than the root causes (Geurts et al., 2024). Accordingly, this study draws upon Conservation of Resources (COR) Theory and incorporates physical activity (PA) and social–emotional competence (SEC) into the analytical framework to reveal the chain mediation mechanism by which digital health literacy (DHL) reduces PMSMU among adolescents (Halbesleben et al., 2014). COR Theory posits that resources possessed by individuals do not exist in isolation but form “Resource Gain Spirals” through continuous investment and transformation; that is, initial resource advantages can further foster new behavioral and psychological resources, thereby enhancing the individual’s capability to cope with environmental stress and risk behaviors (Hobfoll, 1989).

DHL is conceptualized as a critical cognitive resource. Its connotation extends beyond the mere ability to access health information or attitudinal orientations within online environments; rather, it underscores the comprehensive capacity to discern information authenticity, assess risks, and translate relevant information into health behavioral decisions and self-management strategies (Kim and Oh, 2021). Within the highly algorithmic and information-overloaded mobile internet ecosystem, this cognitive resource is particularly pivotal for adolescents in coping with potential issues of excessive use (Firth et al., 2024). Drawing on COR Theory, adolescents possessing relatively sufficient cognitive resources are better equipped to maintain rational judgment when confronted with immediate feedback and emotional reinforcement mechanisms, thereby effectively resisting the onset of problematic usage behaviors (Hobfoll, 2001; Dai et al., 2025). Conversely, adolescents lacking the support of DHL as a core resource often struggle to timely identify potential risks embedded in online content. Furthermore, when facing emotional stimulation and psychological pressure amplified by algorithmic recommendations, they lack the necessary cognitive defenses and self-regulatory capabilities, rendering them more prone to sliding into patterns of maladaptive mobile social network dependence (Xu and Chen, 2025). This theoretical inference is also supported by empirical data; for instance, a study conducted among Chinese university students identified a significant negative correlation between health literacy and PMSMU (Liu et al., 2024). Accordingly, this study further postulates that this relationship holds true within the adolescent population and proposes.

*Hypothesis 1*: DHL is significantly negatively correlated with PMSMU among adolescents.

PA, as a critical behavioral manifestation of a healthy lifestyle, plays a pivotal bridging role in the translation of DHL into real-world health behaviors (Kim and Oh, 2021; Zhu et al., 2025a). On one hand, based on the Knowledge-Attitude-Practice (KAP) Model, DHL not only embodies an individual’s cognitive level regarding health risks such as sedentary behavior but also reflects their capability to utilize digital information and tools to optimize health decisions and implement them in practice (Wu et al., 2022). Adolescents with high DHL are more proficient in utilizing digital technologies—such as health applications and wearable devices—to monitor, obtain feedback on, and adjust their own behaviors, thereby forming a sustained incentive mechanism for PA (Cho et al., 2014; Singh et al., 2025). Simultaneously, acquiring sports skills and scientific exercise knowledge through online platforms helps lower the cognitive thresholds and technical barriers to engaging in PA, thereby enhancing participation motivation and exercise self-efficacy (Hsu et al., 2014; Jiang et al., 2024). On the other hand, according to the Time Displacement Hypothesis, PA, as a structured and goal-oriented offline behavior, competes directly with mobile social network use in terms of time allocation, thereby objectively limiting the opportunities for adolescents to become immersed in mobile social media (Robinson, 1981; Melkevik et al., 2010; Tremblay et al., 2024). Furthermore, from a neurobiological perspective, the “flow experience” and dopamine reward mechanisms induced by exercise can provide more enduring and profound psychological gratification compared to virtual social networks, thereby reducing adolescents’ excessive dependence on the immediate feedback provided by social networks (Elbe et al., 2010; Lynch et al., 2013; Wang et al., 2025). In summary, PA constitutes a vital behavioral pathway through which DHL influences PMSMU. It not only facilitates the transformation of DHL into health behaviors but also indirectly mitigates the risk of PMSMU through mechanisms such as time displacement and psychological gratification. Accordingly, this study proposes:

*Hypothesis 2*: PA plays a mediating role between DHL and PMSMU.

SEC, as a core psychological resource for adolescent psychological development, encompasses multidimensional capabilities such as emotion regulation, interpersonal interaction, and responsible decision-making (Durlak et al., 2011). On one hand, existing studies have confirmed a significant positive correlation between DHL and SEC (Yang et al., 2017; Kim et al., 2023). This association is primarily linked to the mediating mechanism of health self-efficacy; specifically, the sense of control formed during the acquisition and comprehension of health information enhances individuals’ confidence in coping with physical and mental issues. This experience of competence subsequently transfers to social interaction and emotion management contexts, manifesting as more mature modes of emotional expression and interpersonal handling abilities (Hussin and Khan, 2016; Rabenbauer and Mevenkamp, 2021). On the other hand, according to Compensatory Satisfaction Theory, PMSMU can be understood as a maladaptive compensatory strategy adopted by individuals when their real-world psychological needs are not fully satisfied (Bäckman and Dixon, 1992; Kardefelt-Winther, 2014). Adolescents with high SEC are not only more likely to establish stable and high-quality interpersonal connections in real-world contexts, thereby reducing emotional dependence on virtual social interactions (Chen et al., 2021); more critically, their relatively mature emotion regulation and decision-making capabilities enable them to perform effective cognitive reappraisal and impulse control when identifying online temptations or negative emotional triggers, thus lowering the risk of transitioning from normal use to problematic use (Ding et al., 2022). In summary, SEC not only serves as an important manifestation of the spillover effect of DHL but also plays a key psychological regulatory role in inhibiting PMSMU. Accordingly, this study proposes:

*Hypothesis 3*: SEC plays a mediating role between DHL and PMSMU.

**Figure 1:**
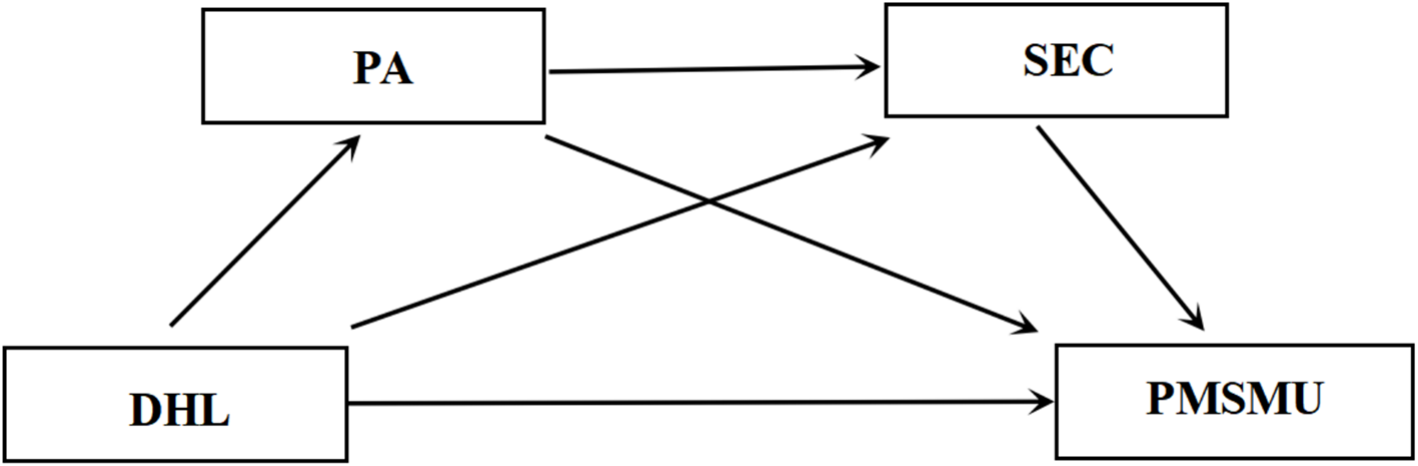
The theoretical model diagram.

PA, owing to its unique social attributes and rich spiritual dimensions involving emotional experience and moral cultivation, serves as a high-value vehicle for cultivating adolescents’ SEC (Fu et al., 2025). Specifically, the inherent rule constraints, goal orientation, and timely feedback mechanisms of PA provide a highly contextualized practice arena for adolescents’ emotional socialization (Tse, 2020). In this process, individuals are required to continuously monitor and regulate their emotions under high levels of physiological arousal, thereby gradually enhancing their emotional adaptability and self-regulation capabilities within complex interactive contexts (Basso and Suzuki, 2016; Zhao et al., 2025). From a developmental psychology perspective, SEC is not an innate trait but rather a capacity that gradually develops through an individual’s active participation in structured social contexts (Opstoel et al., 2020). PA, particularly in team-based or organized forms, provides adolescents with a unique socialization arena in which they encounter authentic situations involving rule constraints, role-taking, cooperation, and conflict resolution in real time. Through such experiences, adolescents gradually internalize and develop core aspects of SEC, including emotion regulation, interpersonal skills, and a sense of responsibility (Smith, 2003; García-Guillén et al., 2025; Liu et al., 2025). In parallel, neurobiological research has further elucidated the physiological mechanisms through which PA supports the development of SEC (Basso and Suzuki, 2016). Regular engagement in PA effectively promotes prefrontal cortex development and modulates the release of key neurotransmitters, including dopamine and serotonin (Lin and Kuo, 2013; Garrett et al., 2024). Specifically, endorphins and dopamine activate the brain’s reward circuitry, eliciting pleasurable experiences, whereas elevated serotonin levels contribute to emotional stability (Lin and Kuo, 2013; Garrett et al., 2024). Together, these neurochemical mechanisms constitute the neural foundation for emotion regulation and impulse control—both of which are core components of SEC (Raji et al., 2025). Taken together, PA not only provides a practical arena for the development of SEC but also establishes the necessary neurobiological foundation for its formation (Ludyga et al., 2022; Zhang et al., 2025a). Accordingly, the temporal precedence of PA over SEC is substantiated by robust evidence from both developmental psychology and neurobiology (Ou et al., 2023; Hill et al., 2024). Notably, the psychological and physiological benefits derived from PA mentioned above are more likely to be sustained and accumulated among adolescents with higher DHL, as this group is more inclined to transform PA into a stable lifestyle and daily habit (Cho et al., 2014; Wu et al., 2022). On this basis, the SEC continuously accumulated through PA can satisfy adolescents’ emotional and social needs in real-world contexts, thereby reducing the likelihood of relying on virtual social networks for compensatory gratification (Chen et al., 2021). Accordingly, this study proposes:

*Hypothesis 4*: PA and SEC play a significant chain mediating role between DHL and PMSMU (see Figure 1).

In summary, grounded in COR Theory, this study constructs a conceptual model with DHL as the predictor and PMSMU as the outcome, specifically examining the independent and chain mediating roles of PA and SEC. Specifically, this study not only investigates the direct impact of DHL on adolescents’ PMSMU but also further examines its indirect effects via the behavioral activation pathway of PA and the psychological regulation pathway of SEC. Furthermore, it explores the hypothesized sequential pathway of “DHL → PA → SEC → PMSMU” as an associative indirect process consistent with theoretical expectations. Through this analytical framework, the study aims to elucidate the potential influencing pathways and underlying mechanisms of adolescent PMSMU from an integrated perspective of cognitive resources, behavioral engagement, and psychological competence development, thereby providing a theoretical basis for relevant intervention practices.

## 2 Methods

### 2.1 Participants

**Figure 2:**
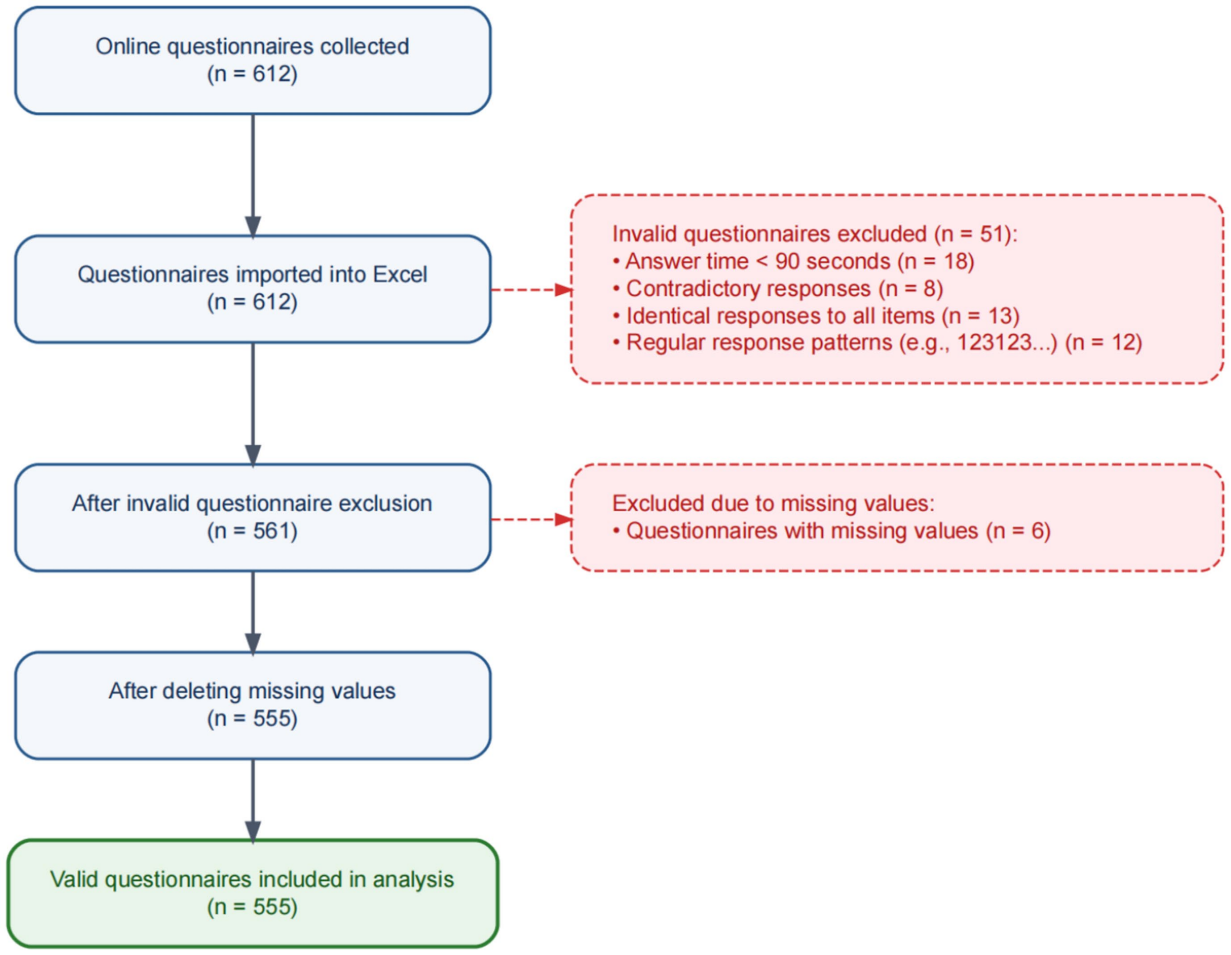
Steps in the screening process for research samples.

Sample size estimation was performed using the Monte Carlo simulation approach developed by Schoemann et al. (2017). Based on 20,000 Monte Carlo simulations with a 95% confidence interval (CI) and a target statistical power of 0.80, the minimum sample size required to detect the hypothesized chain mediation effect was determined to be 436. A convenience sampling method was adopted. Participants were recruited through the online survey platform Sojump,^1^ targeting adolescents aged 10–19 years. To confirm eligibility, an initial screening question regarding age was included in the questionnaire, and only those who met the age criteria were allowed to proceed. Prior to the survey, all participants and their guardians were informed of the study’s purpose, anonymity, and their right to withdraw. Electronic informed consent was obtained from all subjects. The study protocol was approved by the Biomedical Research Ethics Committee of Henan University. A total of 612 online questionnaires were distributed and returned. After data screening, questionnaires were excluded due to short response time (<90 s; *n* = 18), contradictory responses (*n* = 8), identical responses to all items (*n* = 13), and regular response patterns (e.g., straight-lining; *n* = 12). In addition, 6 questionnaires with missing values were removed. Consequently, 555 valid questionnaires were retained for the final analysis, yielding an effective response rate of 90.7%, as shown in Figure 2. The final sample consisted of 300 males (54.05%) and 255 females (45.95%), as shown in Table 1. The participants were aged between 13 and 19 years (mean = 16.20, standard deviations = 1.49).

**Table 1:** Distribution of basic information on adolescents (*N* = 555).

Demographic variables		Number	Proportion%
Age (M±SD)	16.20 ± 1.49	555	100%
Gender	Male	300	54.05%
	Female	255	45.95%

M and SD are used to represent mean and standard deviation, respectively.

### 2.2 Measures

DHL was measured using the eHealth Literacy Scale (Chinese version of eHEALS), translated and revised into Chinese by Guo et al. (2013). The scale consists of eight items assessing individuals’ ability to access, evaluate, and apply online health information and services. All items were rated on a 5-point Likert scale ranging from 1 (strongly disagree) to 5 (strongly agree), with higher scores indicating higher levels of digital health literacy. In the present study, the scale demonstrated good internal consistency (Cronbach’s *α* = 0.80).

PMSMU was assessed using the questionnaire developed by Jiang (2018). The instrument contains 20 items covering five dimensions: increased viscosity, physiological impairment, fear of missing out, cognitive failure, and guilt. Responses were recorded on a 5-point Likert scale, with higher total scores reflecting more severe levels of problematic mobile social network use. In this study, the questionnaire showed good reliability (Cronbach’s α = 0.84).

PA was measured using the Physical Activity Rating Scale–3 (PARS-3), revised by Deqing (1994). The scale evaluates overall physical activity level based on three components: exercise intensity, duration, and frequency. The physical activity score was calculated using the following formula: *Physical Activity Score = Intensity × (Duration − 1) × Frequency.* All items were rated on a 5-point scale, yielding total scores ranging from 0 to 100, with higher scores indicating higher levels of physical activity. The scale exhibited good internal consistency in the present study (Cronbach’s *α* = 0.82). In addition, because the *Physical Activity Score* derived from PARS-3 uses a multiplicative formula that may produce skewed distributions, we performed normality tests to evaluate its distribution. The results showed that although the Kolmogorov–Smirnov and Shapiro–Wilk tests were significant (*p* < 0.001), the skewness (0.945) and kurtosis (−0.139) were within the acceptable range for approximate normality (skewness < |2|, kurtosis < |7|) (Hair et al., 2010). Thus, the PA distribution was considered sufficiently close to normal for parametric analyses.

SEC was assessed using the scale developed by Chen et al. (2023). The scale comprises 26 items across four dimensions: self-related competence, interpersonal competence, group-related competence, and responsible decision-making. Items were rated on a 5-point Likert scale, with higher scores indicating greater levels of social–emotional competence. In this study, the scale demonstrated good reliability (Cronbach’s α = 0.85).

### 2.3 Statistical analysis

Data preprocessing was conducted using Microsoft Excel. Questionnaires with logical inconsistencies, patterned responses, or excessively short completion times were excluded to ensure data integrity and reliability. The cleaned dataset was then imported into the SPSSAU online statistical platform for subsequent analyses (Zhou and Ma, 2024). The analytical procedures were performed as follows. First, descriptive statistics were computed for all major study variables, and Pearson correlation analyses were conducted to examine bivariate associations among variables. Cronbach’s α coefficients were calculated to assess the internal consistency reliability of all measurement scales. Second, Harman’s single-factor test was applied to examine potential common method bias by evaluating whether a single factor accounted for an excessive proportion of the total variance. Finally, a chain mediation model was tested using the bootstrap resampling method (5,000 samples) with 95%CI to examine the mediating effects of PA and SEC in the relationship between DHL and PMSMU.

Prior to the main analyses, the distribution of the age variable was examined. Although the Kolmogorov–Smirnov and Shapiro–Wilk tests were significant (*p* < 0.001), the skewness (0.044) and kurtosis (−1.057) were within the acceptable range for approximate normality (skewness < |2|, kurtosis < |7|) (Hair et al., 2010). Thus, age was considered sufficiently close to normal for parametric analyses.

## 3 Results

### 3.1 Common method bias test

Exploratory factor analysis revealed six factors with eigenvalues greater than 1. The first factor accounted for 17.765% of the total variance, which was below the critical threshold of 40%, indicating that common method bias was not a serious concern in the present study.

### 3.2 Descriptive statistics and correlation analysis

Descriptive statistics and Pearson correlation analyses were conducted for DHL, PMSMU, PA, and SEC. As shown in Table 2, significant correlations were observed among all four variables (all *p* < 0.001), providing preliminary support for the proposed hypotheses. Specifically, DHL was positively correlated with PA (*r* = 0.234, *p* < 0.001) and SEC (*r* = 0.200, *p* < 0.001), and negatively correlated with PMSMU (*r* = −0.191, *p* < 0.001). In addition, both PA (*r* = −0.254, *p* < 0.001) and SEC (*r* = −0.245, *p* < 0.001) were significantly and negatively associated with PMSMU.

**Table 2:** Mean (M), standard deviations (SD), and correlations between the variables.

	M	SD	1	2	3	4
1. SEC	3.779	0.743	1			
2. DHL	3.041	0.802	0.200***	1		
3. PA	30.625	29.302	0.321***	0.234***	1	
4. PMSMU	2.960	0.696	−0.245***	−0.191***	−0.254***	1

*p < 0.05; **p < 0.01; ***p < 0.001.

### 3.3 Chain mediation analysis

As shown in Table 3, after controlling for age and gender, the hypothesized paths remained significant. Specifically, DHL (*β* = −0.117, *p* < 0.01), PA (*β* = −0.173, *p* < 0.001), and SEC (*β* = −0.166, *p* < 0.001) all had significant negative effects on PMSMU. In addition, DHL (*β* = 0.133, *p* < 0.01) and PA (*β* = 0.290, *p* < 0.001) were positively associated with SEC, and DHL was positively associated with PA (*β* = 0.234, *p* < 0.001). Regarding the covariates, gender was significantly associated with SEC (*β* = 0.082, *p* < 0.05), indicating that female adolescents reported higher levels of SEC than their male counterparts. Age was not significantly associated with any of the study variables (*p* > 0.05). The inclusion of these covariates did not alter the significance or direction of the hypothesized mediation effects. These results provide support for Hypothesis 1.

**Table 3:** Hierarchical multiple linear regression analysis results.

Variables	PA		SEC		PMSMU	
	*β*	*t*	*β*	*t*	*β*	*t*
DHL	0.233	5.619***	0.133	3.255**	−0.120	−2.886**
PA			0.294	7.167***	−0.178	−4.110***
SEC					−0.159	−3.706***
Gender	−0.038	−0.908	0.082	2.068*	−0.044	−1.089
Age	0.014	0.340	−0.030	−0.749	0.071	1.767
*R^2^*	0.056		0.127		0.114	
∆*R^2^*	0.051		0.121		0.106	
*F*	10.923***		20.058***		14.085***	

*p < 0.05; **p < 0.01; ***p < 0.001.

**Figure 3:**
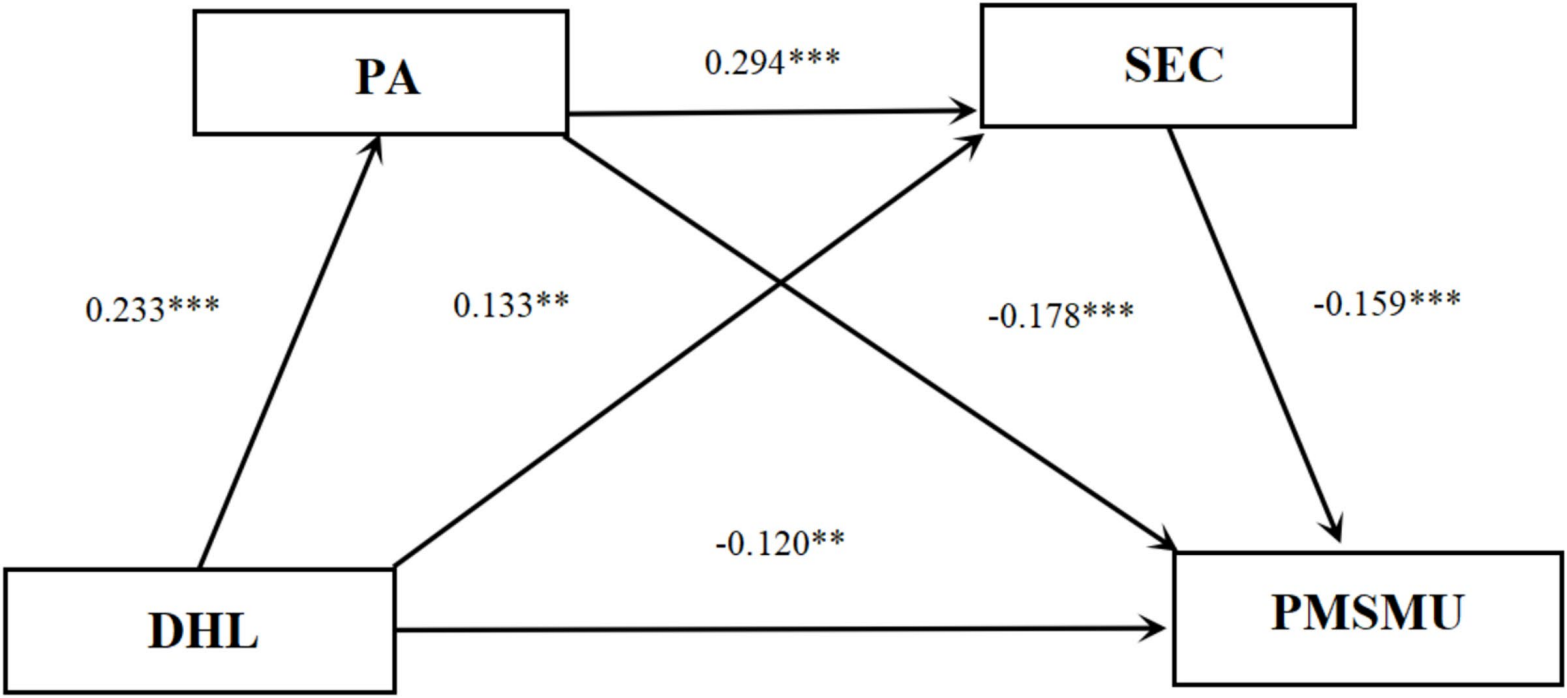
Chain mediation effects of PA and SEC in the relationship between DHL and PMSMU.

As shown in Table 4 and Figure 3, three significant indirect pathways were identified in the mediation model. (1) In Path 1 (DHL → PA → PMSMU), the indirect effect of PA was −0.035, with a 95%CI of [−0.061, −0.016], accounting for 21.43% of the total effect. (2) In Path 2 (DHL → SEC → PMSMU), the indirect effect of SEC was −0.019, with a 95%CI of [−0.034, −0.007], accounting for 10.71% of the total effect. (3) In Path 3 (DHL → PA → SEC → PMSMU), the sequential indirect effect through PA and SEC was −0.010, with a 95%CI of [−0.017, −0.004], accounting for 5.36% of the total effect. Because none of the confidence intervals included zero, all three indirect effects were statistically significant. Therefore, Hypotheses 2, 3, and 4 were supported.

**Table 4:** Direct and indirect effects in the multiple mediator model.

Model	Effect	Boot SE	Boot LLCI	Boot ULCI	Relative mediation effect
Total effect	−0.168	0.036	−0.239	−0.097	100%
Total direct effect	−0.105	0.036	−0.176	−0.033	62.50%
Total indirect effect	−0.064	0.014	−0.094	−0.038	38.10%
DHL ⇒ PA ⇒ PMSMU	−0.036	0.012	−0.061	−0.016	21.43%
DHL ⇒ SEC ⇒ PMSMU	−0.018	0.007	−0.034	−0.007	10.71%
DHL ⇒ PA ⇒ SEC ⇒ PMSMU	−0.009	0.003	−0.017	−0.004	5.36%

Boot SE, Standard error of indirect effects; Boot LLCI, the lower bound of the 95% confidence interval; Boot ULCI, the upper limit of the 95% confidence interval (Percentile Bootstrap Method with Bias Correction).

## 4 Discussion

Grounded in COR theory and informed by a positive psychology perspective, the present study constructed a multiple mediation model to systematically examine the relationship between DHL and PMSMU among adolescents and to elucidate its underlying mechanisms. The results demonstrated that DHL significantly and negatively predicted PMSMU. After incorporating PA and SEC as mediating variables, both the direct and indirect effects of DHL on PMSMU remained significant. It is noteworthy that the overall explained variance in PMSMU was modest (*R^2^* = 0.11), and the sequential indirect effect accounted for only 5.36% of the total effect. Accordingly, caution is warranted when interpreting the present results.

### 4.1 The direct effect of DHL on PMSMU

The present study found that DHL was a significant negative predictor of PMSMU among adolescents, which is consistent with previous empirical findings (Wegmann et al., 2015; Liu et al., 2023; Liu et al., 2024). This result provides further empirical support for the “resource buffering hypothesis” proposed by COR theory, which posits that individuals rely on sufficient psychological resources to cope with environmental stressors—such as information overload and social pressure in digital contexts—in order to reduce the likelihood of maladaptive behaviors (Hobfoll, 1989). As a core cognitive resource, a higher level of DHL reflects not only adolescents’ ability to effectively access and understand health-related information, but also their enhanced health-related self-efficacy (Hussin and Khan, 2016; Rabenbauer and Mevenkamp, 2021). This confidence in one’s capacity for behavioral regulation may encourage adolescents to adopt more adaptive offline lifestyles, thereby reducing their risk of excessive engagement with mobile social networking platforms. In contrast, adolescents with lower levels of DHL often exhibit deficiencies in health information appraisal and risk judgment (Xu and Chen, 2025). Due to difficulties in discerning information quality or accurately evaluating the potential risks associated with excessive social network use, they are more susceptible to algorithm-driven recommendations or biased information, which may foster maladaptive usage patterns. Given that digital health literacy is highly malleable and that prior intervention studies have demonstrated the feasibility and effectiveness of enhancing DHL, incorporating digital health literacy into prevention and intervention frameworks targeting adolescent PMSMU holds clear practical significance and applied value (Wegmann et al., 2015; World Health Organization, 2025c).

### 4.2 The mediating roles of PA and SEC

First, the present findings demonstrate that DHL influences adolescents’ PMSMU through the mediating role of PA, which is consistent with previous studies (Hsu et al., 2014; Yang et al., 2017). From a resource transformation perspective, adolescents with higher levels of DHL are more likely to translate their cognitive advantages in health-related knowledge into sustained engagement in physical activity, thereby achieving an effective conversion of cognitive resources into behavioral resources (Hobfoll, 1989; Li and Ling, 2025). This process aligns with the resource investment principle of COR theory, which posits that individuals tend to deploy existing resources to acquire resources with longer-term returns, thereby reducing the likelihood of psychological resource depletion in high-risk digital environments (Hobfoll, 2001; Li et al., 2023). These findings suggest that merely enhancing health-related knowledge may be insufficient to substantially reduce the risk of PMSMU, whereas sustained activation at the behavioral level plays an indispensable role. In addition, the present results provide further support for the applicability of the time displacement hypothesis within the context of PMSMU research (Robinson, 1981; Melkevik et al., 2010; Tremblay et al., 2024). Specifically, regular participation in PA competes directly with mobile social network use for discretionary time, objectively limiting adolescents’ opportunities for immersion in virtual social environments by reducing non-essential screen exposure, thereby exerting a suppressive effect on PMSMU.

Second, the results indicate that SEC also mediates the relationship between DHL and PMSMU. This finding suggests that the influence of DHL on adolescents’ PMSMU extends beyond health risk awareness alone and operates through deeper pathways related to psychological regulation and social functioning. Specifically, higher levels of DHL may, through adolescents’ sustained interactions with digital environments, gradually strengthen their self-regulatory capacity and social adaptability, producing positive spillover effects across contexts. Prior research has similarly suggested that the accumulation of such cognitive resources can be translated into more mature emotion regulation strategies and more effective interpersonal skills, thereby facilitating the fulfillment of psychological needs in real-life settings (Yang et al., 2017; Kim et al., 2023). From a needs satisfaction perspective, adolescents with higher SEC are more likely to establish stable and high-quality social connections in offline interpersonal contexts, enabling them to obtain sustained social support and a sense of belonging (Bäckman and Dixon, 1992; Kardefelt-Winther, 2014; Zhang et al., 2025b). Compared with the immediate feedback provided by virtual social networks, such real-world psychological satisfaction is superior in both emotional value and stability, thereby reducing adolescents’ motivation to seek compensatory gratification through mobile social network use and, in turn, lowering the risk of problematic use (Wegmann et al., 2025). Moreover, from a self-control and behavioral regulation perspective, higher levels of SEC are typically accompanied by more mature emotion regulation strategies and decision-making abilities (Ding et al., 2022). When adolescents are confronted with emotionally amplified stimuli or usage cues driven by algorithmic recommendations, elevated SEC facilitates proactive management of usage impulses, preventing social network use from escalating unconsciously into excessive or problematic patterns.

The present study further revealed that female adolescents reported significantly higher levels of SEC than their male counterparts, a finding consistent with prior research (Chaplin and Aldao, 2013; Lin et al., 2024; Lee et al., 2025). Relevant studies indicate that, compared with male peers, adolescent girls tend to exhibit more positive emotions during this developmental stage (Chaplin and Aldao, 2013). Specifically, females are more inclined to promote interpersonal harmony, actively initiate interactions with new peers, and demonstrate a stronger tendency to comfort others, which collectively lay a foundation for developing more mature emotion regulation and interpersonal coordination skills (Lee et al., 2025). In parallel, sociocultural factors may reinforce these patterns. Females are often expected to assume caregiving roles and are encouraged to behave in nurturing, expressive, and relationally focused ways, whereas males are more frequently socialized toward instrumental action, assertiveness, and competitiveness (Shek et al., 2021; Lin et al., 2024). Consequently, female adolescents exhibit a more favorable developmental trajectory in emotion recognition, prosocial behavior, and interpersonal management, contributing to their advantage in SEC (Ferreira et al., 2024; Buimer et al., 2025; Lee et al., 2025). Moreover, neurobiological evidence has begun to elucidate the underlying mechanisms (Church et al., 2025). Functional magnetic resonance imaging (fMRI) studies reveal that female adolescents show greater activation in prefrontal cortical regions when regulating emotional responses, supporting the notion that females possess a distinct neural basis for emotion regulation—a core component of SEC (Church et al., 2025). Importantly, the inclusion of gender as a covariate in the statistical model did not alter the significance or direction of the core mediation effects, indicating that the findings are robust.

Finally, the present study further reveals a significant chain mediating effect of PA and SEC in the relationship between DHL and PMSMU. Previous research has consistently identified regular PA as an important antecedent of SEC development (Fu et al., 2025; Yan and Lu, 2025), and the current findings extend this evidence by elucidating its relevance within digital behavior contexts. From a physiological perspective, regular and appropriately structured PA not only enhances adolescents’ physical functioning, but also promotes neurotransmitter release and the development of executive functions in the prefrontal cortex, thereby strengthening self-regulation and inhibitory control and providing a neurobiological foundation for the development of SEC (Lin and Kuo, 2013; Basso and Suzuki, 2016; Kuang et al., 2025). From a social interaction perspective, collective forms of PA offer adolescents frequent and diverse real-world social contexts in which cooperation, communication, and coordination with peers, teachers, and opponents foster continuous practice of social understanding, emotional expression, and interpersonal regulation, thus facilitating the sustained development of SEC (Qian et al., 2025; Yan and Lu, 2025; Zhu et al., 2025b). Notably, in team-based PA, each member assumes clearly defined roles and responsibilities, requiring adolescents to recognize the close linkage between individual performance and collective success. This sense of team belonging encourages adolescents to take responsibility for both their own behaviors and team outcomes during training and competition, thereby strengthening personal and collective responsibility (Bedard et al., 2020). From a developmental psychology perspective, adolescence represents a critical period for social role exploration and rapid development of psychosocial competencies (Fan and Li, 2025). During this stage, stable and continuous engagement in PA provides irreplaceable real-life experiential foundations for the development of SEC. Building on this, the present study further demonstrates that adolescents with higher levels of DHL are more likely to translate their cognitive advantages into sustained participation in PA, and through PA as a concrete behavioral pathway, are associated with the accumulation and reinforcement of SEC. This associative pattern is consistent with a sequential indirect pathway linking DHL to PMSMU. Nevertheless, given the cross-sectional design of the present study, causal interpretations of this sequential order should be made with caution, and longitudinal research is needed to verify the hypothesized temporal sequence.

### 4.3 Implications and limitations

The present study systematically examined the relationship between DHL and PMSMU, revealing that adolescents with higher levels of DHL are more likely to translate cognitive resources into real-world engagement in PA, which in turn promotes the development of SEC and ultimately reduces the risk of PMSMU. These findings provide a more dynamic and integrative explanatory framework for understanding adolescents’ problematic digital behaviors. From a theoretical perspective, this study contributes to a deeper understanding of the antecedents and underlying mechanisms of PMSMU and offers important insights for psychological intervention and behavioral guidance among adolescents. Grounded in positive psychology and COR theory, the present research clarifies the relationship between DHL and PMSMU and elucidates the mediating roles of PA and SEC. By shifting the focus beyond risk-oriented explanations of PMSMU, the findings highlight the internal logic through which adolescents’ existing strengths and protective resources can be activated to counteract problematic digital behaviors, thereby enriching the theoretical framework for explaining PMSMU. From a practical perspective, the findings provide valuable implications for educators, parents, and mental health professionals in developing more constructive intervention strategies. The results suggest that interventions targeting adolescent PMSMU should not be confined to restrictive management approaches or solely cognitive education, but should place greater emphasis on the systematic cultivation of digital health literacy and the activation of healthy lifestyle behaviors. Specifically, educational practices may focus on enhancing adolescents’ abilities to access and critically evaluate digital health information, thereby guiding them toward more informed and adaptive health-related decision-making. At the same time, coordinated efforts between families and schools can be leveraged to encourage regular participation in PA and to intentionally foster SEC within PA contexts. Such a comprehensive intervention model—integrating cognitive, behavioral, and psychological resource development—may help adolescents build a stable and enriched reservoir of real-world psychological resources, thereby fundamentally reducing their emotional reliance on virtual social networks.

Despite these contributions, several limitations of the present study should be acknowledged. First, the study constructed its analytical model using overall scale scores rather than examining individual sub-dimensions of each measure. Treating constructs as global indicators, rather than disaggregating their constituent dimensions, may yield different interpretations of the underlying mechanisms. Future research is therefore encouraged to explore the unique roles of specific sub-dimensions within each scale. Second, the cross-sectional design of this study limits causal inference among the variables. Although the theoretical framework and statistical findings support the proposed chain mediation model, longitudinal designs or experimental intervention studies are needed to further verify the long-term causal relationships among DHL, PA, SEC, and PMSMU. Third, the reliance on self-reported data may introduce potential biases related to social desirability or recall. Future studies could incorporate objective behavioral indicators or multi-informant assessment methods to enhance the robustness and validity of the findings. Finally, the use of a convenience sampling approach via an online survey platform may limit the representativeness of the sample and introduce selection bias. Although efforts were made to recruit participants from diverse provinces across China, the non-probability sampling method means the findings may not be fully generalizable to the broader adolescent population. Future research should consider probability sampling strategies to enhance sample representativeness and external validity.

## 5 Conclusion

Using a chain mediation model, the present study systematically examined the mechanisms through which DHL influences PMSMU among adolescents. The results demonstrated that DHL was negatively associated with PMSMU, both directly and indirectly, through the independent and sequential mediating roles of PA and SEC. These findings suggest that interventions aimed at reducing adolescent PMSMU should move beyond singular approaches focused solely on knowledge transmission or risk awareness. Instead, more effective and sustainable intervention strategies may be achieved by adopting a multi-level framework that simultaneously targets cognitive, behavioral, and psychological functioning—specifically by promoting adolescents’ engagement in PA while concurrently fostering their SEC.

## Data Availability

The original contributions presented in the study are included in the article/supplementary material, further inquiries can be directed to the corresponding author.

## Footnotes

1
www.sojump.com
